# The Relationship between Microstructure and Mechanical Properties of PBST Two-Component Crystalline Random Copolymers with Different BT Contents

**DOI:** 10.3390/polym15020383

**Published:** 2023-01-11

**Authors:** Mingjun Gang, Yuanxia Wang, Yu Zhang, Lizhi Liu, Ying Shi

**Affiliations:** 1 Advanced Manufacturing Institute of Polymer Industry, Shenyang University of Chemical Technology, Shenyang 110142, China; 2 School of Materials Science and Engineering, Shenyang University of Chemical Technology, Shenyang 110142, China

**Keywords:** poly (butylene succinate-co-butylene terephthalate), microstructure, mechanical properties

## Abstract

The mechanical properties of two-component crystalline random copolymers are primarily based on their microstructure. At the same time, the influence of the composition on the crystallization behavior and crystal structure of these materials is also well known. Thus, in this study, a poly (butylene succinate-co-butylene terephthalate) random copolymer (PBST) with different molar ratios of butylene terephthalate (BT) was prepared. A systematic analysis of the crystallization behavior, crystal structure, and mechanical properties of PBST with different BT contents was carried out using WAXD, SAXS, and DSC analyses. The investigations showed that PBST-37.5 containing 37.5 mol% of BT content had the lowest strength and highest elasticity among the different compositions. This was because the two-component crystallization of poly (butylene terephthalate) (PBT) and poly (butylene succinate) (PBS) was greatly inhibited at the corresponding BT composition and the crystal growth was the least perfect, imparting poor strength to the PBT-37.5. Alternately, when the content of BT was 32.5 mol% in the PBST, the PBS segment could crystallize, and both PBT and PBS crystals were formed in the PBST-32.5. Thus, PBST-32.5 showed a higher material hardness than PBST-37.5. In contrast, when the BT content was greater than 37.5 mol% in the PBST, only PBT crystals existed in the PBST copolymer. Further, as the BT content increased, the crystal size of PBT gradually increased, which led to a closer packing of the crystal arrangement, increasing the crystallinity. This led to a gradual increase in the strength of the PBST material and a gradual decrease in its elasticity.

## 1. Introduction

At present, an efficient method of obtaining biodegradable polymers with excellent mechanical properties is the copolymerization of aliphatic and aromatic units [1,2,3]. A two-component crystalline biodegradable polymer, poly (butylene succinate-co-butylene terephthalate) (PBST), can be synthesized from poly (butylene terephthalate) (PBT) and poly (butylene succinate) (PBS) by random copolymerization [4,5]. Because the PBT chain segment has a stronger crystallization capacity, changing the BT content of the PBST copolymer can make a huge difference in the performance of PBST [6,7]. Extensive research has been carried out on studying the characteristics of PBST at higher BT contents of above 50 mol%, and the PBST-70 copolymer has been found to show a strong crystallization ability. In addition, PBT chain segments provide good mechanical strength and processability for PBST copolymers [8,9]. Although PBST copolymers with a low BT content (30–50 mol%) exhibit excellent shape memory, room temperature processing, and other special properties, reports on the impact of low BT contents on the characteristics of PBST copolymers are scanty [10,11]. Therefore, it is worthwhile to systematically study the relationship between the crystallization behavior, crystal structure, and mechanical properties of PBST with a low BT content for developing PBST copolymers with functional applications.

The crystalline structure of biphasic crystalline random copolymers is relatively complex, and the crystallization behavior of copolymers with different components and compositions varies greatly. Therefore, understanding and regulating the crystallization behavior of biphasic copolymers is of immense significance for designing and developing materials with appropriate properties [12,13,14]. At present, a considerable number of studies have been carried out on the synthesis and performance characterization of PBST materials. Many researchers have reported on the synthesis of PBST random copolymers, of which PBS–PBT multiblock copolymers were successfully synthesized by blending PBS and PBT (containing 31–87 mol% PBT) [5,6,15,16]. Additionally, various PBST-branched copolymers with long-branched chains were also reported [3,17]. Nevertheless, more attention has been drawn toward the structure and properties of linear random PBST copolymers due to their biodegradability, physical properties, and processing properties, which depend more on the BT content in the PBST. Specifically, PBST materials with a higher BT content have always attracted much attention in the study of linear random PBST copolymers [18,19]. It is widely reported that the structure and properties of PBST-70 copolymers with a 70 mol% BT content can be used in fiber materials through melt spinning [20,21,22,23,24]. Since PBT crystals are the main crystals in PBST-70, solving the influence of the slow crystallization rate and unstable crystallization morphology of PBT crystals on fiber manufacturing has gained significant attention. Subsequently, the mechanical properties of PBST-70 have been improved, and the crystallization rate of PBST was accelerated by adding inorganic nanofillers such as cyclodextrin polymer (CDP), organically modified layered zinc phenylphosphonate (m-PPZn), silica, and fibrous attapulgite nanoparticles [19,25,26]. Studies on PBST-70 copolymer further revealed that the influence of strain on the lamellar structure is much greater than that of temperature during the stretching and heating process. Moreover, the formation of a fibrous crystal structure was observed under tension above 100 °C with no obvious PBS crystals in the structure [18,27]. A lower crystallinity of PBST copolymers with a decreasing BT content was evident from the literature. When the BT content is higher than 50 mol%, the stronger BT segment predominantly inhibits the crystallization ability of the soft BS segment [6,28]. Similarly, the PBS chain segment in PBST-50 copolymers (at 50 mol% BT) can crystallize very slowly at room temperature, forming a tassel-like micelle structure in the PBT crystal network, enormously improving the mechanical properties of the material [20]. In contrast, the performance of PBST copolymers greatly varies with a 35 mol% BT content. In PBST-35 copolymers, only the PBT chain segment can crystallize at room temperature. However, a higher proportion of PBS components enhances the crystallization capacity of PBS significantly upon stretching. Interestingly, the PBS crystals, during the stretching process, form a temporary-shaped switch due to the memory effect, making PBST-35 a degradable memory plastic [10]. On the other hand, PBS crystals dominate in copolymers with a 10 mol% BT content, and the crystal diffraction pattern changes gradually from PBS to PBT crystals as the BT content approaches 30 mol% [6,28].

The crystalline structure of biphasic crystalline random copolymers made of PBST material has its own unique characteristics, and the crystalline structure changes dramatically at different BT contents. Obviously, these changes in crystal structure induce differences in the performance of PBST copolymers [15,29]. However, systematic research on the structure and mechanical properties of PBST copolymers with low BT contents of 30–50 mol% is still limited. Since the properties of the materials have shown considerable changes within the range of 30–50 mol% BT contents, differences in the crystallization behavior of biphasic copolymers are anticipated, which presents completely different application directions. Therefore, the present work focused on the synthesis of random copolymers of PBST with low BT contents and the relationship between the crystalline structure of biphasic PBST copolymers and different BT contents, and the synergistic effects of the copolymers and the material properties were all explored. This study can lay a good foundation for the development of PBST materials for different functional applications.

## 2. Materials and Methods

### 2.1. Materials

The PBST random copolymers with different BT contents were synthesized by the Sinopec research team. The synthesis was carried out by melt polycondensation of 1,4-butanediol (BDO: AP degree, Beijing Yili Fine Chemicals Co., Ltd., Beijing, China), purified terephthalic acid (PTA: AP degree, Beijing Chemical Reagent Co., China), and succinic acid (SA: AP degree, Sanyi Chemicals Co., Ltd., Shenzhen city, China.) using rare-earth compounds (purity of 99.5%, Beijing Xinhua Reagent Factory, Beijing, China.)as catalysts. The PBST random copolymers with weight-averaged molecular weights (M_w_) of about 10 × 10^4^ g/mol were synthesized and measured by gel permeation chromatography (GPC)with chloroform as the eluent. The detailed synthesis procedure can be found in the cited literature [30]. The sample names of the PBST copolymer with specific BT contents are detailed in Table 1.

### 2.2. Differential Scanning Calorimetry (DSC) Analysis

The thermal properties of the blends were analyzed by a thermal analysis (TA) instrument (Q100 system, Newcastle, DE). Approximately 5–6 mg of the sample was sealed in an aluminum sample holder and heated under a nitrogen environment from room temperature to 200 °C at a rate of 40 °C/min. The system was then maintained at a constant temperature for 5 min to eliminate the thermal history of the material. Later, it was cooled to −40 °C at a rate of 10 °C/min and again maintained at a constant temperature for 5 min. Finally, the temperature was raised to 200 °C at a rate of 10 °C/min. The analysis of the crystallization, crystallinity, and glass transition temperature of the copolymer was performed using DSC.

### 2.3. Small-Angle X-ray Scattering (SAXS)

To evaluate the microstructures and periodic structures of the materials, SAXS measurements (Beijing, China) were carried out in the Beijing Synchrotron Radiation Facility (BSRF). The PBST copolymers were made into 2 mm thick plates by a flat vulcanizer at 120 °C in order to form test samples of the PBST copolymers for the SAXS measurements. The SAXS characterization was performed using a λ = 0.1542 nm synchrotron for synchrotron acceleration at a beamline of 1W2A. The sample detector was set at a distance of 1520.20 mm in the beam direction of the SAXS data collection. The exposure time was fixed to 10 s.

### 2.4. Wide-Angle X-ray Diffraction (WAXD)

The PBST copolymers were made into a 2 mm thick plates by a flat vulcanizer at 120 °C in order to form test samples of the PBST copolymers for the WAXD measurements. The WAXD measurement (Beijing, China) was carried out using a Bruker (Germany) D8 DISCOVER 2D ray diffractometer. An IµS-type generator was operated at 40 kV and 650 µA, and Cu radiation with a 0.1542 nm wavelength was used for the experiment. The 2D X-ray diffraction patterns were collected with a VÅNTEC-500 detector with a pixel size of 68 × 68 µm^2^ and a beam spot size of 0.5 mm. The distance from the sample to the detector was 95 mm, and the exposure time was 300 s.

### 2.5. Tensile Testing

The PBST samples were made into 2 mm thick plates for tensile testing by a flat vulcanizer at 120 °C, and they were then cut into pieces to make the tensile samples. The tensile test was carried out at room temperature with an Instron 5965 instrument (Instron Shanghai, China). Dumbbell-shaped 5A samples with an initial length of 20 mm were used for the tensile tests, which were performed at a speed of 50 mm/min. The test procedure was conducted according to ISO 527–2:1993.

### 2.6. Hardness Testing

A hardness study was conducted with a Zwick/Roell Digi Test 3115 (Shanghai, China) instrument equipped with a Shore A detector. The PBST samples were made into 2 mm thick plates for hardness testing by a flat vulcanizer at 120 °C. Testing was conducted according to the ISO 868 standards.

## 3. Results and Discussion

### 3.1. Study on the Microstructure of PBST by WAXD

**Figure 1:**
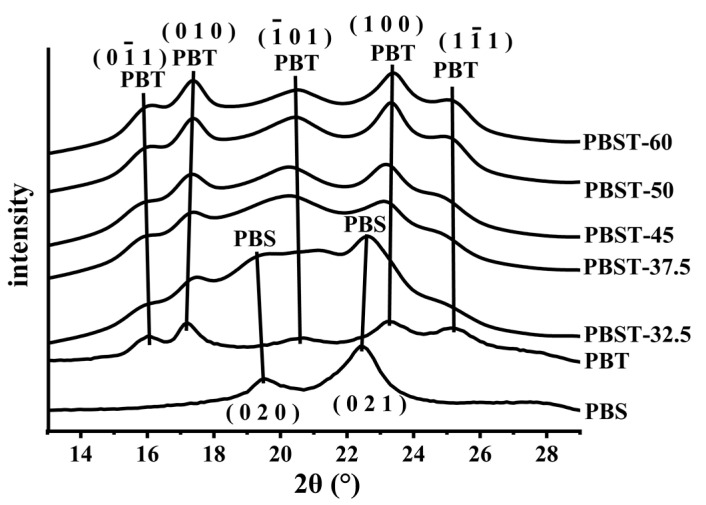
The diffraction profiles of neat PBT, PBS homopolymers, and PBST copolymers with the BT content.

The crystalline structure of the PBST samples were analyzed by WAXD. The WAXD curves of the PBS homopolymer, PBT homopolymer, and PBST copolymers with different BT contents are shown in Figure 1. The comparison of the diffraction profiles of all three samples revealed that the characteristic peaks of the PBS homopolymer at 19.2° and 22.4° could be observed in the PBST-32.5 copolymer diffraction profile. Furthermore, the PBT homopolymer’s characteristic peaks at 16.1°, 17.2°, 20.2°, 23.2°, and 25.2° could be clearly observed in the PBST-32.5 copolymer (Figure 1). This indicated that the PBS and PBT crystals existed simultaneously in the PBST-32.5 copolymer. This was very similar to the crystallization behavior of poly (butylene succinate-co-adipate) (PBSA) [31]. Moreover, the diffraction peak of PBS in the PBST-32.5 shifted to a lower angle (19.2°) when compared to neat PBS (19.4°) [10]. According to Bragg’s equation, when the wavelength of the incident ray was unchanged, the decrease in the diffraction angle (θ) indicated that the distance between the crystal planes (d) increased. Therefore, it can be inferred that the PBS crystal in the PBST-32.5 was more loosely packed than that of the neat PBS homopolymer, which indicated that the degree of order of the PBS crystals in the PBST-32.5 was lower than that in the neat PBS homopolymer.

In contrast, the characteristic peaks of PBT were only present in the diffraction profiles of all four samples when the BT content was higher than 32.5 mol% in the PBST, indicating that the PBS chain segments could no longer crystallize in the material at this time. This can be explained based on the crystallization capacity of the PBS chain segment, which was far lower than that of the PBT chain segment. At a lower BS content, the length of the PBS chain segment decreased, reducing the formation of mature crystals. It can be seen that the angles of the characteristic peak of PBT of the four PBST copolymers (PBST-37.5, PBST-45, PBST-50, PBST-60) were lower than that of the pure PBT homopolymers. For example, the peak position of the diffraction peak on the crystal plane of the pure PBT crystal (1 -1 -1) was 25.3°, the characteristic peak in PBST-32.5 was 25.2°, the characteristic peak in PBST-37.5 was 25°, the characteristic peak in PBST-45 was 24.9°, and the characteristic peaks in the PBST-50 and PBST-60 copolymers were 24.9°. Therefore, the peak position of the diffraction peak on the crystal plane of PBT crystal (1 -1 -1) in the PBST copolymer was lower than that on the crystal plane of PBT crystal (1 -1 -1) in the pure PBT homopolymer [8]. This suggested that the averaged inter-distance between neighboring crystals in PBST-37.5, PBST-45, PBST-50, and PBST-60 was larger than that in PBST-32.5 and thus formed a looser crystal structure. Alternately, the presence of the BS segment in the copolymer led to a decreased length of the PBT chain segment. The lower chain segment length of PBT restricted the growth of PBT crystals, resulting in the formation of low-order PBT crystals [19].

According to the Scherrer formula (D = Kλ/βcosθ, where D is the crystal size along the normal direction of a given crystal plane, K is a shape factor (0.89), λ is the wavelength of the X-ray (0.154 nm in our case), β is the full width at half-maximum intensity (FWHM) of a diffraction peak in radians, and θ is the diffraction angle.) and Bragg’s equation (d = Kλ/2sinθ, where d is the crystal plane spacing) [32,33], the crystal size and the crystal plane spacing for the PBST copolymers were calculated based on the diffraction peak at 17.3° and are depicted in Table 2 (because the diffraction peak at 17.3° was the least affected by the other diffraction peaks). For the four copolymers (PBST-37.5, PBST-45, PBST-50, and PBST-60), the increasing BT content in the PBST copolymer increased the PBT crystal size, and the crystal plane spacing of PBT crystals decreased. This suggested that the PBT chain segments were stacked more closely with increasing the BT content, and the crystal size became more and more perfect. Therefore, increasing the BT content in the PBST resulted in longer PBT chains being produced. The longer the PBT chain segment, the easier it was for the molecular chains to be stacked in order and the easier it was for the PBT crystal to grow.

### 3.2. Crystallization Behavior of the PBST Copolymers

**Figure 2:**
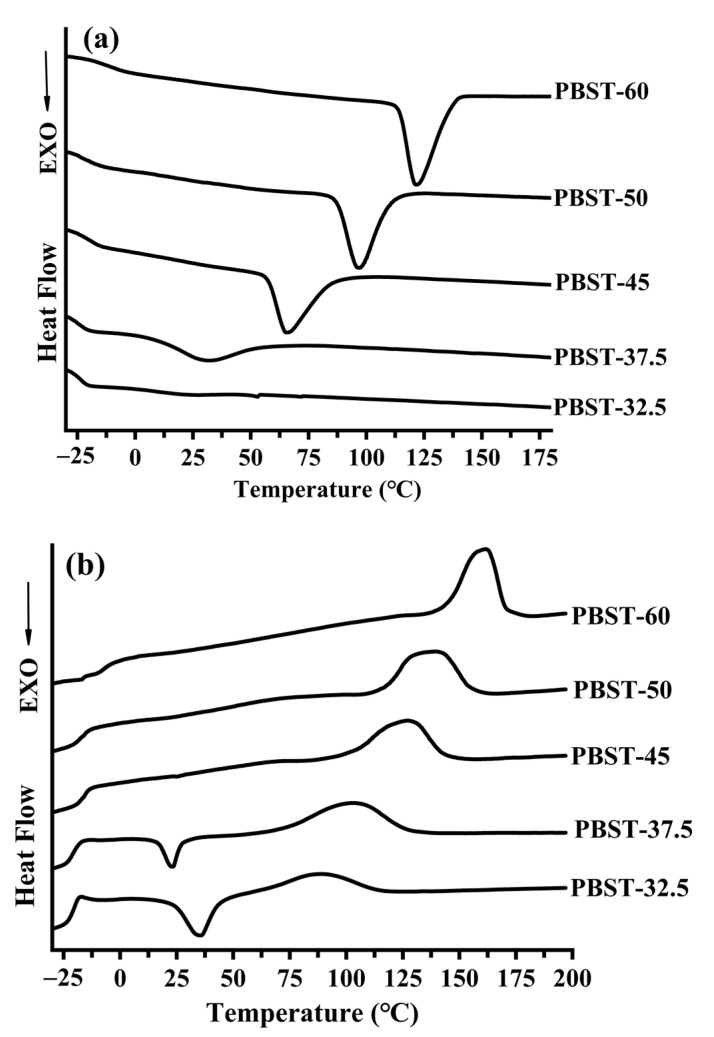
The heating curve of PBST copolymers with different BT contents: (**a**) primary cooling of PBST copolymers; (**b**) secondary heating of PBST copolymers.

The crystallization behavior of the PBST copolymers with varying BT content was investigated by DSC, and the corresponding thermal performance curves are shown in Figure 2a. As can be observed from the curves, a single glass transition temperature (T_g_) was recorded for the PBST copolymers, and it lay between the T_g_ of the PBS homopolymer (31 °C) and PBT homopolymer (40.5 °C) [15.29]. This indicated that the PBST copolymer was a random copolymer containing a combination of short PBS and PBT chain segments [11].

The T_g_ of the PBST gradually shifted towards the T_g_ of the PBT homopolymer with increasing the BT content in the PBST. This can directly infer the influence of the PBT chain segment on the T_g_ of the PBST copolymer. As the BT content in PBST increased, the PBS segments became shorter; thus, the impact of the BS segments on the PBST molecular chains was reduced considerably. Therefore, the higher the BT content in the PBST, the shorter the PBS segments, and the lower the BS segments affected the PBST’s molecular chains. The crystallization of the PBT segment in the PBST gave rise to crystallization peaks in the PBST-37.5, PBST-45, PBST-50, and PBST-60 samples (Figure 2a). The crystallization temperature of the PBST rose with the gradual increase in the BT content, indicating a stronger crystallization ability of the PBST. Further, increasing the BT content in the copolymer made the length of the PBT segment longer, and thus the PBT segments were more likely to form regular stacking structures. The crystallization peak areas of the PBST-32.5 and PBST-37.5 copolymers were very small, indicating a poor crystallization capacity, which was further supported by the temperature rise curve shown in Figure 2b [6,34,35].

As shown in Figure 2b, the crystallization ability of the PBST-32.5 and PBST-37.5 samples was very weak, and thus the crystallization process could be completed at the rate of 10 °C/min during the cooling process. The cold crystallization peak of the PBST-32.5 and PBST-37.5 copolymers was found near 30 °C [36,37]. At the same time, it was observed that the cold crystallization temperature of PBST-37.5 was lower than that of PBST-32.5, indicating that the molecular chains in the PBST-37.5 sample could move even at lower temperatures to form crystals. This was due to the presence of crystals grown during the previous cooling step of the PBST-37.5 sample, and these already-existing crystals could act as nuclei for the cold crystallization process [38,39,40,41]. These crystals improved the crystallization ability of the PBST-37.5 sample, making the cold crystallization temperature of PBST-37.5 lower than that of PBST-32.5. Therefore, the crystallization capacity of PBST-37.5 was greater than that of PBST-32.5. On the other hand, when the content of BT in the PBST exceeded 37.5 mol%, the cold crystallization peak near 30 °C disappeared, indicating that the PBST could complete its crystallization process during the cooling process itself. Thus, the crystallization ability of the PBST increased with increasing the BT content in the PBST [3]. Similarly, the melting temperature of the PBST copolymer crystal at a BT content higher than 37.5 mol% was higher than that of the PBS homopolymer crystal (112.9 °C), which indicated that the crystals formed by the PBST with a BT content above 37.5 mol% corresponded more with the PBT crystals [11]. Considering the conclusions from the WAXD and DSC analyses, it can be confirmed that only the PBT chain segments could crystallize in the random copolymer at a BT content greater than 37.5 mol%. The heating curves of the PBST copolymers (Figure 2b) with increasing the BT content showed an increased melting temperature and a sharp melting peak, suggesting the formation of perfect crystals. This was attributed to the increase in the chain length and the regularity of the PBT molecular chain in the PBST copolymer [21,42,43]. According to the formula X_c_ = ∆H_m_/M_PBT_ • ∆H_m_^0^, where ∆H_m_ is the melt detail of PBT in the PBST copolymers, ∆H_m_^0^ is the standard melt detail of pure PBT, ∆H_m_^0^ = 144.5 J/g, and M_PBT_ is the molar content of PBT in the copolymer, the crystallinity of the PBT chain segments in the PBST copolymer is shown in Table 3. Because there were two kinds of crystals, PBT and PBS, in the PBST-32.5 copolymer but there was only one melting peak in the DSC curve, it was impossible to accurately separate the proportion of the melt detail PBT and PBS crystals by the DSC curve. Therefore, the crystallinity of PBT in the PBST-32.5 copolymer was not calculated in this paper. The crystallinity of the PBT chain segments in the PBST copolymer was enhanced with increasing the content of BT in the copolymer at a BT content higher than 32.5 mol%. This can be ascribed to the increasing length of the PBT chain segment, the reduced crystallization process of the PBT chain segment in the PBST by the influence of the BS segment, and the easier formation of the regular stack structure of the PBT chain segment with the increasing the BT content in the PBST. Further, this was consistent with the conclusion of the WAXD analysis.

### 3.3. Study on the Lamellar Structure of PBST by SAXS

**Figure 3:**
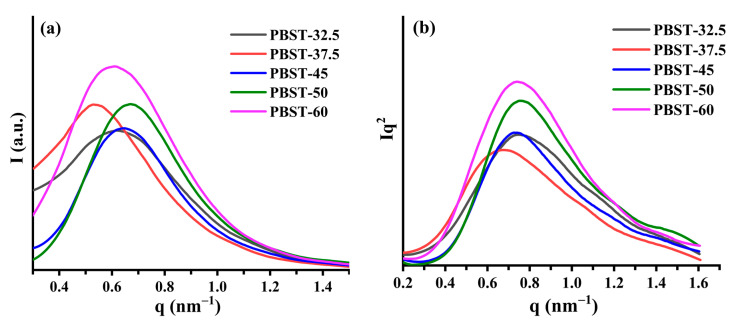
The SAXA curve and Lorentz-corrected SAXS profiles for the PBST with different BT contents. (**a**) The SAXA curve; (**b**) Lorentz-corrected SAXS profiles.

The nanostructure of the PBST was analyzed using SAXS analysis. It can be observed from Figure 3 that the scattering peak position of the PBST-37.5 copolymer was lower than that of the PBST-32.5 copolymer. The long period (L_p_) of the PBST copolymer structure with different BT contents was calculated using the equation Lp = 2π/q_max_, where q_max_ is the scattering vector, by Lorentz-corrected SAXS profiles. The thickness of the amorphous layer was calculated according to the correlation function [31]. The thickness of the lamella was calculated by the L_p_ minus the thickness of the amorphous layer (shown in Table 4). It was found that the L_p_ of the PBST-37.5 copolymer was greater than that of the PBST-32.5 copolymer, which may be due to the long period (L_p_) being related to the lamellar thickness and the thickness of the amorphous layer. The lamellar thickness and the amorphous layer thickness of PBST-37.5 were higher than those of PBST-32.5. This may be because the PBST-37.5 sample contained more mobile BS segments, which can favor the crystallization of BT. Additionally, the scattering peak half-peak width of PBST-32.5 was larger than that of PBST-37.5. This can be ascribed to the presence of PBS and PBT crystals and the superposition of the lamellar structure signals of the PBS and PBT crystals in the PBST-32.5 sample.

When the BT content exceeded 37.5mol% in the PBST, the peak position of the PBST showed a negligible change with the increase in the BT content, indicating that the L_p_ change in the PBST was not significant (see Table 4). However, the diffraction peak intensity of the PBST increased with the increase in the BT content in the PBST, suggesting an increase in the lamellar crystal content of PBT in the PBST. This can be ascribed to the fact that with the increase in the BT content in the PBST, the influence of the BS segment on the PBT segment molecular chain length was reduced. Therefore, the PBT chain length continued to grow, more PBT chain segments were arranged orderly in the system, and the lamellar crystals became thicker. These results were well in agreement with the conclusions drawn from the DSC and WAXD analyses.

### 3.4. Study on Mechanical Properties of PBST

**Figure 4:**
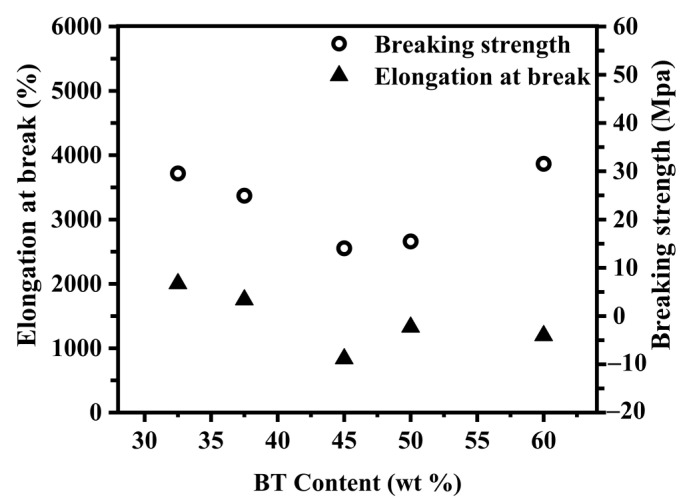
The elongation at break and breaking strength of PBST copolymers with different BT contents.

To explore the influence of the PBST structures with different BT contents on their mechanical properties, tensile testing and hardness testing were carried out on the samples. Figure 4 shows the changes in elongation at break and the breaking strength of the PBST copolymers with different BT contents. The elongation at break represents the ratio of displacement at fracture to the original length of the specimens of the PBST copolymers with different BT contents, reflecting the change in the toughness of the PBST copolymers. As can be observed from Figure 4, the elongation at break of the PBST copolymers showed an overall downward trend with increasing the BT content in the PBST. This was because the toughness of the BS segment was better than that of the BT segment, and the PBST copolymers were strongly dependent on the BS segment content. Therefore, with the increase in the BT content and the decrease in the BS content in the PBST copolymers, the elongation at break of the PBST copolymers showed an overall downward trend. Meanwhile, the PBST-45 sample had the lowest breaking strength. Because of the low content of BS and the short chain length of PBS, the PBST-45 was difficult to be induced to crystallize during stretching. Moreover, the formed PBT crystals inhibited the formation of PBS crystals during the stretching process, leading to a low PBS crystal content. The low strength of the PBS crystals had no significant contribution toward the strength improvement in the material, and thus the PBST-45 sample had the lowest breaking strength at different BT contents in the PBST copolymers. The breaking strength of the PBST sample continued to increase with the decrease in the BT component content below 45 mol% because of the increasing BS component. The higher BS content enhanced the ability to induce PBS crystallization during the tensile process, thus improving the breaking strength of the material. [10] In contrast, the breaking strength of the material continued to increase with the increase in the BT content above 45 mol%. This can be explained as increasing the BT content raised the high-strength PBT crystal content gradually in the PBST, which led to the continuous enhancement of the fracture strength of the material [11].

**Figure 5:**
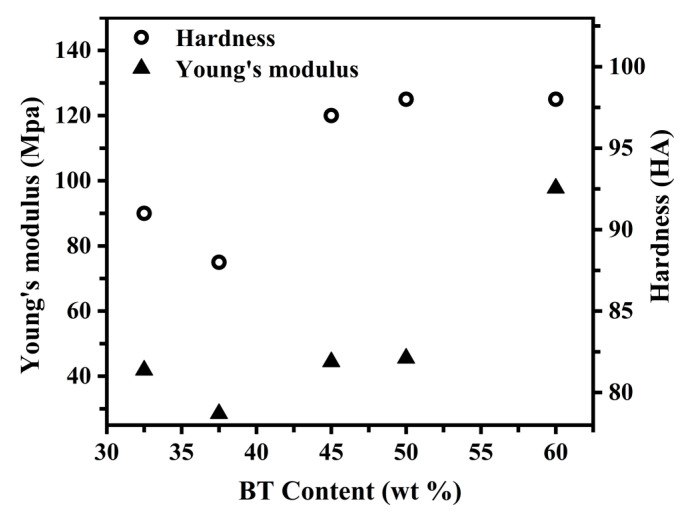
Young’s modulus and hardness of PBST copolymers with different BT contents.

As can be observed from Figure 5, the value of Young’s modulus and the hardness of the PBST copolymers with different BT contents showed similar trends. Among all, PBST-37.5 had the lowest Young’s modulus and hardness. This was because the inhibition of the PBS and PBT two-component crystallization was the largest in PBST-37.5; thus, the crystal content of the PBST-37.5 sample was low, making the material weak in resistance to external deformation. Interestingly, it can be seen in Figure 5 that the value of Young’s modulus and the hardness of PBST-32.5 were higher than that of PBST-37.5. Since the PBS content in the PBST-32.5 sample was higher, the degree of the mutual crystallization inhibition of the PBS and PBT was smaller than that of PBST-37.5. This resulted in the existence of both PBS and PBT crystals in the PBST-32.5 sample, increasing the crystal content, which enhanced the resistance to external deformation. On the other hand, the value of Young’s modulus and the hardness of the PBST copolymers increased with the increase in the BT content above 37.5 mol%. This can be attributed to the gradual increase in the content of high-strength PBT crystals with increasing the BT content in the PBST, gradually enhancing the deformation resistance of the material.

**Figure 6:**
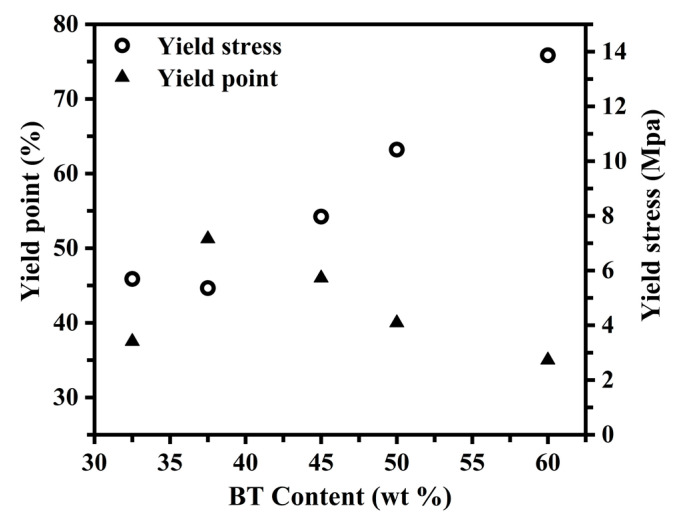
The yield point and yield stress of PBST copolymers with different BT contents.

Figure 6 shows the change in the yield point and yield stress of the PBST copolymers with different BT contents. The yield point of the PBST copolymer represents the degree of deformation before the plastic deformation of the material begins. In other words, it is the degree of deformation before the crystal in PBST begins to be fragmented, and it represents the elastic capacity of the material. The PBST-37.5 sample had the highest yield point and the largest elasticity because it had the largest degree of mutual crystallization inhibition between the PBS and PBT components as well as the lowest crystallinity. The PBST-37.5 sample could be easily deformed due to the smallest limiting effect of the PBST-37.5 crystals on the deformation of materials. On the other hand, the lower yield point of PBST-32.5 compared to that of PBST-37.5 was because of the lower inhibition degree of the two-component crystallization of PBS and PBT than that of PBST-37.5, resulting in the formation of both PBS and PBT crystals in the PBST-32.5 sample, both of which had a limiting effect on the deformation of the material. At the same time, the low strength of PBS crystals can fragment the crystals even under low stress, and thus, the degree of deformation of the PBST-32.5 sample occurred before plastic deformation and was lower than that of PBST-37.5. Similarly, when the content of BT was higher than 37.5 mol%, it was found that the degree of deformation of the PBST copolymer decreased gradually before plastic deformation with the increase in the BT content. This was because the increasing BT content raised the PBT crystal content in the PBST, gradually decreasing the size of the amorphous region in the material and strengthening the limiting effect of the crystals on the deformation of the PBST material.

The yield stress of the PBST copolymer reflects the stress at the beginning of destruction in the PBST copolymer crystals with different BT contents. The crystal structure of the PBST-37.5 copolymer had the smallest stress at the beginning of destruction because the PBS and PBT crystals in the PBST-37.5 same had the highest degree of mutual inhibition with the least perfect crystal growth and a smaller crystal size, resulting in the destruction of the crystal in the PBST-37.5 sample even under low stress. However, the crystals of the PBST-32.5 sample required a slightly greater stress than the PBST-37.5 sample when it began to fragment. This can be explained based on the existence of both PBS and PBT crystals in PBST-32.5, both of which enhanced the strength of the material. However, the low-strength PBS crystals were fragmented even under small stress, and thus the yield stress of the PBST-32.5 sample was not significantly improved. On the other hand, the stress required for the crystals of the PBST copolymers to begin to fragment gradually increased with increasing the BT content above 37.5 mol%. This was because of the increase in the PBT crystal content, which has a larger strength.

## 4. Conclusions

The present work described the effect of crystal behavior and crystal structure on the mechanical properties of PBST copolymers prepared with different BT contents. The structural analysis of the PBST material was carried out using DSC, SAXS, and WAXD analyses. The systematic investigation of the crystallization behavior and crystal structure of the PBST copolymers with different BT contents drew the following conclusions:

(1) When the BT content was 37.5 mol% in the PBST, the mutual inhibition of the PBS and PBT segments was most obvious, which led to the formation of only small-sized PBT crystals in the PBST-37.5 copolymer. These copolymers were composed of loosely packed crystal stacks and exhibited the lowest crystallinity.

(2) When the content of BT was 32.5 mol% in the PBST, the higher content of the BS component favored the crystallization of the PBS component. Owing to the strong crystallization ability of the PBT component, both PBT and PBS crystals were formed in the PBST-32.5 sample.

(3) When the BT content in the PBST was greater than 37.5 mol%, the PBT chain segments became longer with increasing the BT content and were easily arranged into an ordered structure. Therefore, the PBT crystal size increased gradually, and the crystallinity of the PBT increased continuously.

(4) When the content of BT was 37.5 mol% in the PBST, the inhibition of the two-component crystallization of the PBS and PBT was the largest, the crystal growth was the most imperfect, and the crystal content was lower, resulting in a lower yield stress and Young’s modulus than that of the PBST-32.5 sample. However, the yield point was higher in the PBST-37.5 sample than that in the PBST-32.5 sample, indicating that PBST-37.5 had higher elasticity than PBST-32.5.

(5) The crystallinity of PBT continued to rise, and the strength of the PBT crystals became higher with increasing the BT content above 37.5 mol%. This resulted in an increasing trend in the yield stress and hardness of the PBST, while the yield point decreased, and the elasticity was weakened.

(6) During the process of stretching, the breaking strength of the PBST-45 containing 45 mol% of BT content was found to be the lowest. Although the PBS component of the PBST was induced to crystallize, the inhibition effect of the PBT crystals suppressed PBS crystal formation. This resulted in a lower crystallization content of PBS in the PBST-45 sample. In addition, the lower strength of PBS crystals led to their easy deformation during the stretching process, imparting the lowest breaking strength to the PBST-45 sample.

Conclusively, this work systematically studied the relationship between the microstructure and mechanical properties of PBST two-component crystalline random copolymers with different BT contents. The present study provides deeper insights for understanding and regulating the crystallization behavior of biphasic polymers and thus is of immense importance in designing and guiding the development of material properties.

## Figures and Tables

**Table 1:** The sample names and specific BT contents of PBST samples.

Sample	BT (mol %)	BS (mol %)
PBST-32.5	32.5	67.5
PBST-37.5	37.5	62.5
PBST-45	45	55
PBST-50	50	50
PBST-60	60	40

**Table 2:** Crystal size and crystal plane spacing of PBST copolymers with different BT contents when the diffraction peak was at 17.3°.

	PBST-37.5	PBST-45	PBST-50	PBST-60
Crystal size(nm)	10.3	10.6	12.5	13.5
Crystal plane spacing (nm)	0.514	0.512	0.511	0.511

**Table 3:** Crystallinity of PBT in PBST copolymers with different BT contents.

	PBST-37.5	PBST-45	PBST-50	PBST-60
X_c_ (%) ^1^	9.33	10.3	12.1	12.6

^1^ Xc is crystallinity of PBT in PBST copolymers.

**Table 4:** The long period (L_p_) of PBST copolymer with different BT contents.

	PBST-32.5	PBST-37.5	PBST-45	PBST-50	PBST-60
L_p_ (nm) ^1^	8.39	9.38	8.64	8.34	8.44
Lamellar thickness (nm)	1.89	2.78	2.04	2.04	2.14
Amorphous layer thickness (nm)	6.5	6.6	6.6	6.3	6.3

^1^ L_p_ is long period of PBST copolymer.

## Data Availability

All data generated or analyzed during this study are included in this published article.
